# Multimodal Secondary Prevention Behavioral Interventions for TIA and Stroke: A Systematic Review and Meta-Analysis

**DOI:** 10.1371/journal.pone.0120902

**Published:** 2015-03-20

**Authors:** Maggie Lawrence, Jan Pringle, Susan Kerr, Joanne Booth, Lindsay Govan, Nicola J. Roberts

**Affiliations:** 1 Institute for Applied Health Research, Glasgow Caledonian University, Glasgow, United Kingdom; 2 School of Education, Social Work and Community Education, University of Dundee, United Kingdom; 3 Health Economics and Health Technology Assessment, Institute of Health and Wellbeing, University of Glasgow, Glasgow, United Kingdom; Medical University Innsbruck, AUSTRIA

## Abstract

**Background:**

Guidelines recommend implementation of multimodal interventions to help prevent recurrent TIA/stroke. We undertook a systematic review to assess the effectiveness of behavioral secondary prevention interventions.

**Strategy:**

Searches were conducted in 14 databases, including MEDLINE (1980-January 2014). We included randomized controlled trials (RCTs) testing multimodal interventions against usual care/modified usual care. All review processes were conducted in accordance with Cochrane guidelines.

**Results:**

Twenty-three papers reporting 20 RCTs (6,373 participants) of a range of multimodal behavioral interventions were included. Methodological quality was generally low. Meta-analyses were possible for physiological, lifestyle, psychosocial and mortality/recurrence outcomes. Note: all reported confidence intervals are 95%. Systolic blood pressure was reduced by 4.21 mmHg (mean) (−6.24 to −2.18, P = 0.01 I^2^ = 58%, 1,407 participants); diastolic blood pressure by 2.03 mmHg (mean) (−3.19 to −0.87, P = 0.004, I^2^ = 52%, 1,407 participants). No significant changes were found for HDL, LDL, total cholesterol, fasting blood glucose, high sensitivity-CR, BMI, weight or waist:hip ratio, although there was a significant reduction in waist circumference (−6.69 cm, −11.44 to −1.93, P = 0.006, I^2^ = 0%, 96 participants). There was no significant difference in smoking continuance, or improved fruit and vegetable consumption. There was a significant difference in compliance with antithrombotic medication (OR 1.45, 1.21 to 1.75, P<0.0001, I^2^ = 0%, 2,792 participants) and with statins (OR 2.53, 2.15 to 2.97, P< 0.00001, I^2^ = 0%, 2,636 participants); however, there was no significant difference in compliance with antihypertensives. There was a significant reduction in anxiety (−1.20, −1.77 to −0.63, P<0.0001, I^2^ = 85%, 143 participants). Although there was no significant difference in odds of death or recurrent TIA/stroke, there was a significant reduction in the odds of cardiac events (OR 0.38, 0.16 to 0.88, P = 0.02, I^2^ = 0%, 4,053 participants).

**Conclusions:**

There are benefits to be derived from multimodal secondary prevention interventions. However, the findings are complex and should be interpreted with caution. Further, high quality trials providing comprehensive detail of interventions and outcomes, are required.

**Review Registration:**

PROSPERO CRD42012002538.

## Introduction

Stroke, a chronic, debilitating condition, is projected to remain one of the leading causes of death and adult disability for the foreseeable future [1]. Annually, approximately 15 million people worldwide have a stroke; approximately one third will die and one third will be left permanently disabled [1]. Without intervention, it is projected that, globally, deaths caused by stroke will rise to 7.8 million in 2030 [2]; the number of living stroke survivors is expected to rise to 77 million [2]. Following transient ischaemic attack (TIA) or stroke, rates of recurrence are high: 8.1% within 48 hours following TIA [3], and at 10 years following stroke the cumulative risk of recurrence is 39.2% [4]. Such high rates of recurrence indicate the need for early implementation of effective secondary prevention measures that address modifiable risk factors including: hypertension, abnormal blood lipids, smoking, diet, physical activity, alcohol consumption, and psychosocial stress and depression [5]. Evidence-based clinical guidelines recommend implementation of multimodal approaches to secondary prevention [6,7] i.e. complex interventions that address all of the following: prescription of appropriate medication in conjunction with active provision of information and education regarding stroke, lifestyle (behavioral) risk factors, and medication adherence. To enhance effectiveness and maximise the potential for patient compliance, it is further recommended that such interventions be informed by behavior change theory and make use of behavior change techniques, such as motivational interviewing [8].

While there is a strong impetus for the implementation of multimodal interventions, understanding of the effectiveness of such interventions and the processes involved in supporting the initiation and subsequent maintenance of behavior change is limited [9]. Previously published literature reviews have been narrow in scope, or have focussed only on particular elements of intervention e.g. exercise [9, 10–13]. Consequently, there remains a gap in knowledge regarding the effectiveness of multimodal interventions. Therefore, to begin to address this evidence gap, we undertook a systematic review to determine the effectiveness of multimodal secondary prevention interventions following TIA and/or stroke. Future work will focus on unpicking the detail of the prcesses and mechanisms of action.

## Methods

The review was conducted as described in a protocol registered with PROSPERO (CRD42012002538; S1 Text) using Cochrane Collaboration methods [14], and is reported here in accordance with PRISMA guidelines [15] (S1 PRISMA Checklist). All screening, extraction and assessment processes were conducted by two of four reviewers (ML, JP, SK, JB) working independently; any disagreements were resolved by consensus, with arbitration by a third reviewer, if necessary.

### Inclusion criteria

We determined inclusion criteria relating to Participants, Interventions, Comparator and Outcomes (PICO; S1 Box) [16]; all included studies were randomized controlled trials (RCTs). Study participants were required to be adults aged ≥18 years who had had a stroke. A broad definition of stroke was adopted, to include ischaemic stroke, haemorrhagic stroke, subarachnoid haemorrhage and TIA [17] (Hanto, 1976). In terms of intervention, stroke secondary prevention interventions were required to be ‘multimodal’. Multimodal was defined as a complex intervention which addresses: 1) medication education and/or medication compliance education; 2) education or active information provision e.g. about stroke, stroke (lifestyle) risk factors; and 3) one or more of four specified lifestyle behaviors i.e. smoking, diet, physical inactivity, and alcohol consumption, and/or behaviors associated with amelioration of lifestyle risk factors i.e. medication compliance and management of perceived psychosocial stress. Comparator was usual care or modified usual care e.g. a schedule of phone calls that mimics the schedule of ‘intervention’ calls made to the intervention group. Primary outcomes of interest included physiological outcomes e.g. blood pressure, blood lipids, and lifestyle behavior change. Secondary outcomes of interest were psychosocial outcomes e.g. anxiety, learning outcomes e.g. knowledge of lifestyle risk factors for stroke, and incidence of vascular events and mortality. The latter were described as secondary outcomes rather than primary outcomes because, despite their clinical importance, few studies report long-term follow-up data, 3–6 months being the most frequently reported follow-up period.

### Database and Search Strategies

In January 2014, searches were conducted in a comprehensive range of electronic databases i.e. AMED, ASSIA, CINAHL, Cochrane Central Register of Controlled Trials, Cochrane DARE, DORIS, Embase, ERIC, EThOS, Health Management Information Consortium, Medline, PsycINFO, Social Services Abstracts, and ZETOC. Selected medical subject headings (MeSH) were combined with keywords relating to stroke, secondary prevention, and specific lifestyle behaviors i.e. smoking, physical inactivity, diet, alcohol and perceived psychosocial stress, to create a search strategy, finalised for use in MEDLINE (S2 Text), and amended for use in the other databases, using appropriate controlled vocabulary, Boolean operators and search symbols. Delimiters were: dates searched (1980–2014); research subjects (human); and language (English). In addition, we scanned the reference lists of relevant papers, including systematic reviews, for potentially relevant studies. Bibliographic management software, RefWorks, was used to store and manage the results of the database searches.

### Data Extraction and Quality Assessment

Data, including details of study design and methods, study populations, interventions (delivery and content), and primary and secondary outcomes, were extracted from papers using a data extraction tool adapted for this review from our earlier review [11] (S3 Text). Authors were contacted to request provision of any missing data. Methodological quality was assessed using the Cochrane Risk of Bias tool [14]. Quality was assessed as being of low/unclear/high risk of bias against seven criteria: random sequence generation (selection bias), allocation concealment (selection bias), blinding of assessors (performance bias), blinding of outcome assessment (detection bias), incomplete outcome data (attrition bias), selective reporting (reporting bias), and ‘other’.

### Data analysis

We calculated the differences between intervention and control groups, post-intervention. Continuous outcome measures were expressed as mean value post treatment in each group and variances were derived from standard deviations. These data were analysed using fixed effects inverse-variance meta-analysis for difference in means between intervention and control groups with 95% confidence interval (CI). The fixed effect model is the best model to use if (a) there is reason to believe that all the studies are functionally identical, (b) our goal is to compute the common effect size, which would then be generalized to other examples of this same population, (c) there are no studies with extreme effect sizes that could influence the results, or (d) the number of studies is very small, meaning it may be difficult to estimate the between-studies variance (the extra variance added in the random effects model) with any precision. It is reasonable to assume that the studies included in our analysis are estimating the same effect size, and our goal is to compute this common effect.

Where post treatment outcomes were measured more than once, we used the later measurement. To determine long-term effectiveness of interventions, we conducted sub-analysis for the follow-up point for which most data were available i.e. 12 months. Dichotomous outcomes were analysed using fixed effects Mantel-Haenszel meta-analysis with odds ratios (ORs) and 95% CI. Heterogeneity was assessed using I^2^; heterogeneity >50% was considered noteworthy [14]. We used Review Manager v.5.1 [18] to perform our statistical analyses. The focus of secondary prevention intervention is on initiation of behaviors that will help to reduce the risk of recurrence of stoke and other cardiovascular events, with the aim of sustaining the benefits of intervention in the long-term. Therefore meta-analyses were conducted on all available data, irrespective of data collection time point and sub-analyses were conducted where 12-month data were available. Note: all data are reported with 95% CIs.

## Results

### Description of Included Trials

**Fig 1:**
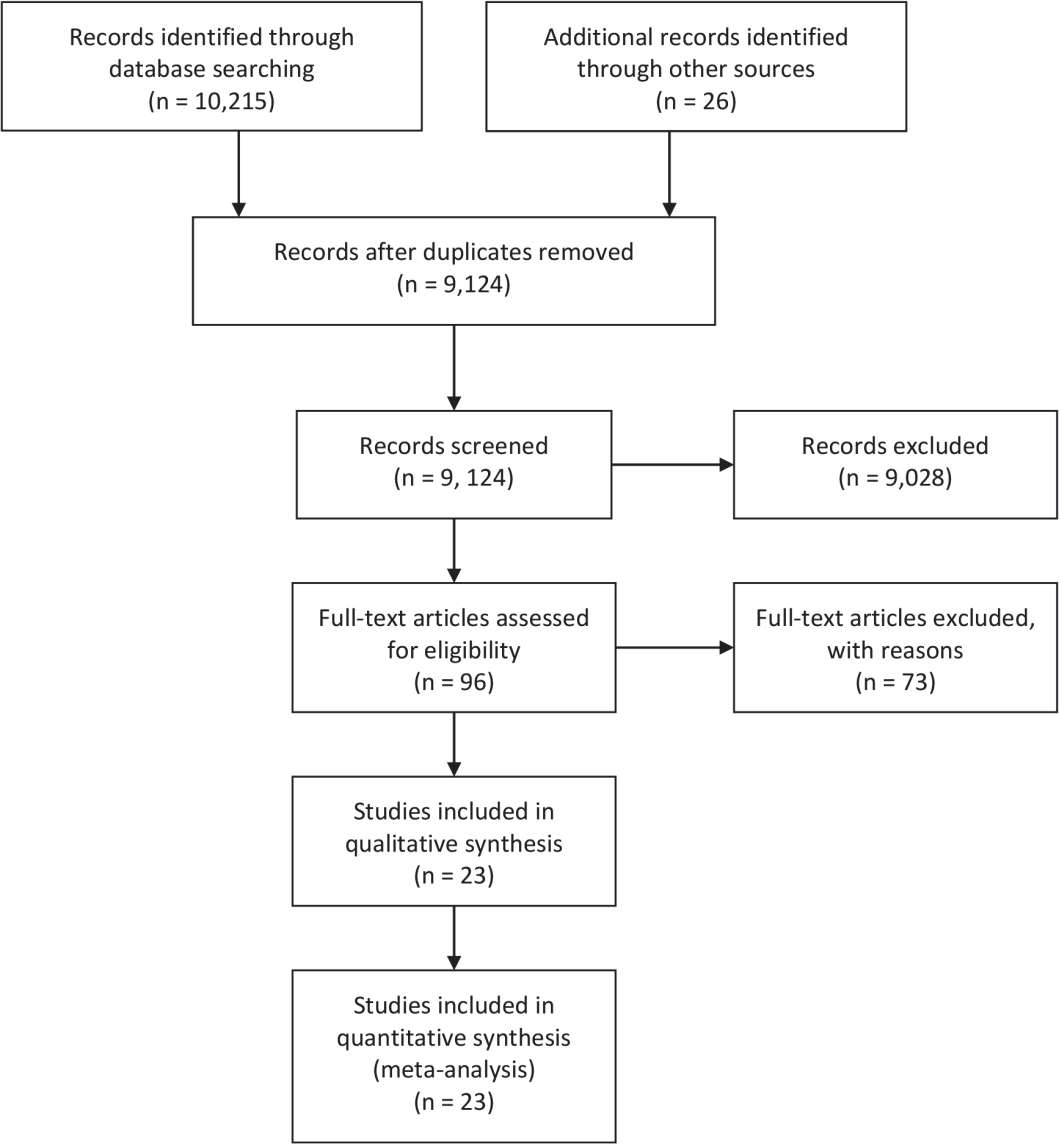
Details of the Flow of Papers through the Review Process.

Database searches identified 9,098 unique bibliographic references. Review of title and abstracts resulted in the exclusion of 9,028 papers that did not meet the broad inclusion criteria (TIA/stroke; RCT). Full texts were retrieved for the remaining 70 papers, and for an additional 26 papers identified by the grey literature searches (i.e. n = 96). These papers were screened for eligibility using the detailed PICO criteria. This resulted in the exclusion of 73 papers; 23 papers were included in the review (Fig. 1). Papers were rejected because they were not RCTs (n = 24), included populations other than stroke (n = 1), were not multimodal secondary prevention interventions (n = 22), or did not report relevant outcomes (n = 26).

The 23 papers reported 20 RCTs. Extracted data from the 23 papers are presented in the evidence table (Table 1), however not all of these papers reported data in a format that facilitated inclusion in the meta-analyses reported below. Ellis et al., (2005) and McManus et al., (2009) reported the same nurse-led study, Ellis et al., at three-month follow-up, McManus et al., at 36-months, therefore, for the purposes of this review, they were considered as one paper [19,20]. Faulkner et al., (2103a) and Faulkner et al., (2013b) both reported HEPAP, an exercise-based intervention [21,22]. Intervention details were extracted from both papers; results were taken from the 2013b paper [22]. Goldfinger et al., (2012) and Horowitz et al., (2013) reported the same community-based intervention (PRAISE) [23,24]. Intervention details were extracted from both papers; previously unpublished results were provided by the authors [25].

**Table 1:** Evidence table: participant details and study characteristics.

Authors, Year, Country	Study participants	Intervention type Theory/Model	Intervention initiation, frequency, duration	Data collection times Outcomes of interest	Completers; Significant results at final follow-up
Adie & James 2010 UK	I: n = 29, Male: 12; Age: 73.6 (SD 8.0); C: n = 27, Male: 16; Age: 71.2 (SD 9.7)	1 to 1; Telephone-based education, advice & counseling; Social Cognitive Theory	7–10 days post-stroke; Initial telephone counseling (one-off session) & then at 1, 2 & 4 months post-stroke	Baseline & 6 months; BP, cholesterol, change in medication knowledge, smoking, diet, exercise	Completers: I: 29; C: 27 I: non-significant reduction in cholesterol, significant medication knowledge; no other significant results
Allen et al., 2009 USA	I: n = 190, Male: 91 Age: 68 (SE 1) C: n = 190; Male: 99 Age: 69 (SE 1)	1 to 1; Care management approach; Chronic illness model	In-home assessment ≤1 week of discharge; Telephone contact weekly (1 month), then monthly (6 months); home visits as required	Baseline & 6 months BP, cholesterol, HbA1c, lifestyle modification, stroke knowledge, QoL	Completers: I: 163; C: 175; I: significant effect on lifestyle modification (p = 0.0003) and stroke knowledge (p = 0.0003); no other significant results
Banet & Felchlia, 1997; USA	I: n = 28, Male: NR Age: NR; C: n = 28, Male: NR; Age: NR	1 to 1; Patient-held shared medical records & education pack Theory: NR	At discharge Individual supported to record behavioral goals & keep records updated; followed up for 6 months	Baseline & 6 months Miller’s Intention Scale & Behavior Scale for diet, smoking & exercise	Completers: I: 24; C: 28 No significant results
Chanrueng-vanich et al., 2006 Thailand	I: n = 31, Male: 10 Age: 62.8 (SD 7.4); C: n = 31, Male: 10; Age: 63.2 (SD 7.1)	Group education then self-regulation; Social Cognitive Theory & Health Promotion Model	≥ 6 weeks post-stroke; 12-week education & exercise programme	Baseline, 6 & 12 weeks; HR, BP, fibrinogen, cholesterol, physical activity questionnaire	Completers: I: NR; C: NR; No significant results
Damush et al., 2011 USA	I: n = 87, Male: NR; Age: NR; C: n = 87, Male: NR; Age: NR	Format: NR; Social Cognitive Theory & self-management	≤ 1 month post-discharge Telephone support biweekly for 12 weeks	Baseline, 3 & 6 months SSQoL, Self-Management Behavior Frequency, medication compliance	Completers: I & C: 123; No significant results
Eames et al., 2013 Australia	I: n = 71 (31 carers, 40 pats), Male: 38; Age: 55.2 (SD 16.7); C: n = 67 (30 carers, 37 patients), Male: 31 Age: 61.4 (SD 12.7)	1 to 1/dyad Computer-generated tailored information booklet & verbal reinforcement Health Belief Model & principles of adult learning	Recruited prior to discharge; Monthly phone calls for 3 months	Baseline & 3 months Stroke Knowledge, self-efficacy, SA-QoL, Anxiety	Completers: I: 60; C: 59 I: improved self-efficacy for accessing stroke information (p = 0.004) & feeling informed (p = 0.008); There were no other significant results
Ellis et al., 2005 / McManus et al., 2009 UK	I: n = 49, Male: NR Age: NR; C: n = 53, Male: NR; Age: NR	1 to 1/dyad; Health education & counselling Theory: NR	≤ 3 months post-stroke; Counselling interviews, monthly for 3 months	Baseline, 5 months & 3.6 years; BP, HbA1c, cholesterol, smoking, QoL, depression, survival	Completers: I: 49: C: 53; No significant results
Faulkner et al., 2013a/ Faulkner et al., 2013b; New Zealand	I: n = 33, Male: 16 Age: 68 (SD 11) C: n = 37, Male: 15 Age: 69 (SD 10)	Group exercise & education Individual exercise prescription; Health Belief Model	≤ 2 weeks post-onset; 2 sessions per week for 8 weeks: 90 minutes exercise, 30 minutes education	Baseline, 2 & 3 months BP, HbA1c, cholesterol, BMI, waist circumference, smoking	Completers: I: 30; C: 30 I: significant improvement in systolic BP (p = <0.5); no other significant results
Flemming et al., 2013; USA	I: n = 20, Male: 10 Age: 73.3 (SD 13) C: n = 21, Male: 14 Age: 71.0 (SD 9)	1 to 1; Education, goal planning, motivational interviewing; Theory: NR	< 12 weeks post-onset; Visits at baseline, 6 weeks, 6 & 12 months; Phone calls at 3 & 9 months	Baseline, 6 & 12 months; BP, HbA1c, cholesterol, BMI, physical activity frequency, alcohol & tobacco use, diet	Completers: I: 18; C: 18; I: significant improvement in systolic LDL (p = 0.0083); no other significant results
Gillham & Endacott, 2010; UK	I: n = 26, Male: NR Age: 67.7 (SD 12.0); C: n = 26, Male: NR; Age: 68.9 (SD 13.2)	1 to 1; Education & support; Transtheoretical model	Time post-stroke: NR; Initial interview then MI telephone follow-up at 2 & 6 weeks	Baseline & 3 months HADS, Readiness to change, alcohol, smoking, exercise, diet	Completers: I: 25; C: 25 I: significant improvement in self-reported exercise (p = 0.007) & diet (p = 0.033); no other significant results
Goldfinger et al., 2012 (protocol)/ Horowitz et al., 2013/Negron et al. 2014; USA	I: n = NR, Male: NR Age: NR; C: n = NR, Male: NR Age: N	Community groups; PRAISE (Prevent Recurrence of All Inner-city Strokes through Education) Education & self-management (peer-led); Theory: NR	1.8 years (SD 1.5) Weekly workshops for 6 weeks	Baseline & 6 months BP, LDL, weight, BMI, medication compliance, smoking, alcohol, knowledge, HRQoL, stress	Completers: I: 242; C: 266; I: significant improvement in systolic BP and diastolic BP
Hornnes et al., 2011 Denmark	I: n = 172, Male: 76 Age: 70.2 (SD 13.7); C: n = 177, Male: 79; Age: 68.5 (SD 12.2)	1 to 1; PREVENT (Post-Stroke Preventive Trial) Education, counselling; Behavioral counseling	Pre-discharge or at first OPD appointment Home visits at 1, 4, 7 & 10 months	Baseline & 1, 4, 7 & 12 months; BP, medication compliance, recurrent event	Completers: I: 145; C: 158; I: significant improvement in BP (p = 0.007); no other significant results
Joubert et al., 2006 Australia	I: n = 46, Male: 23 Age: 64.7 (SD 14.9); C: n = 51, Male: 25; Age: 68.2 (SD 12.5)	1 to 1/dyad Integrated shared-care model Theory: NR	Post-discharge GP visits at 2 weeks, 3, 6, 9, & 12 months Telephone assessment prior to each visit; information to GP	Baseline & 12 months BP, cholesterol, blood glucose, BMI, exercise, smoking, alcohol	Completers: I: 35; C: 45 I: significant improvement in cholesterol (p = 0.02) & exercise (p = 0.048); no other significant results
Joubert et al., 2009 Australia	I: n = 123, male: 53 Age: 63.4 (SD 13.7); C: n = 110, Male: 49; Age: 68.2 (SD 12.7)	1 to 1/dyad ICARUSS (Integrated Care for the reduction of Secondary Stroke); Theory: NR	Post-discharge GP visits at 2 weeks, 3, 6, 9, & 12 months Telephone assessment prior to each GP visit; information sent to GP	Baseline, 3 & 12 months; BP, BMI, cholesterol, alcohol, smoking, exercise, stroke knowledge, QoL	Completers: I: 91; C: 95 I: significant improvement in BMI (p = 0.007) & exercise (p<0.001); no other significant results
Kirk et al., 2013; UK	I: n = 12, Male: 9 Age: 67.5 (SD 11.4); C: n = 12, Male: 10; Age: 66.8 (SD 7.3)	Group; Education & exercise Theory: NR	One month post-event; Weekly classes for 6 weeks (adapted) Cardiac Rehabilitation Programme	Baseline & 5 months BP, BMI, waist-hip ratio	Completers: I: 12; C: 12 I: significant improvement in activity levels (p = 0.029); no other significant results
Kono et al. 2013; Japan	I: n = 35, Male: 21 Age: 63.5 (SD 7.0); C: n = 35, Male: 27; Age: 63.4 (SD 11.4)	1 to 1; Exercise training, advice & counseling; self-education Behaviour change theory	Post-discharge Weekly exercise training for 24 weeks & self-education; advice & counseling baseline 3 & 6 months	Baseline, 3 & 6 months Recurrent event, BP, cholesterol, HbA1c, weight, BMI, daily step counts, daily salt intake, diet, smoking, alcohol	Completers: I: 34; C: 34 I: significant decrease in sBP (p< 0.001), significant increase in HDL (p = 0,022) & daily physical activity (p = 0.012), & significant decrease in salt intake (p<0.001); no other significant results
Maasland et al. 2007 The Netherlands	I: n = NR, Male: 17 Age: 63 (SD 13); C: n = NR, Male: 17; Age: 65 (SD 12)	Individualized COSTA: computer-supported health education; Theory: NR	Time since onset: NR; Individualized multi-media computer programme Frequency: at individual’s discretion	Baseline & 3 months BP, cholesterol, weight smoking, alcohol, exercise, medication compliance, stroke knowledge	Completers: I: 27; C: 30 No significant results
Peng et al. 2014 China	I: 1795, Male: n = NR; Age: 61.5 (SD 11.5); C: n = 2026, Male: n = NR; Age: 60.3 (SD 11.7)	1 to 1; Medication, lifestyle advice, education (computer software); Theory: NR	Time since onset: NR; Frequency: NR; Duration: NR	Baseline, 6 & 12 months; Recurrent event; Medication compliance; Programme adherence	Completers: I: 1287; C: 1430; I: significant compliance with statins (p = 0.006); no other significant results
Sit et al. 2007; Hong Kong, China	I: n = 107, Male: 55 Age: 62.8 (SD 10.3); C: n = 83, Male: 50; Age: 64.0 (SD 12.0)	Group; Education, group work & individualized goal setting; Self-management	Time since onset: NR; Group meetings (2 hours) weekly for 8 weeks	Baseline & 3 months; BP, cholesterol, smoking, alcohol, diet, exercise, medication compliance, stroke knowledge	Completers: I: 77; C: 70 I: significant improvements in diet (p = 0.004), self-monitoring of BP, stroke knowledge & medication compliance (all p< 0.001); C group significantly reduced exercise; no other significant results
Wolfe et al. 2010; UK	I: n = 274, Male: 148; Age: 20% >80 years; C: n = 249, M: 131; Age: 22% >80 years	Individualized; Stop Stroke, tailored risk management Theory: NR	10 weeks; Algorithm applied at 10 weeks, 5 & 8 months	Baseline & 12–18 months; BP, smoking, alcohol, medication compliance	Completers: I: 273; C: 247; No significant results

Of the 20 trials (6,373 participants at baseline), four were conducted in Asia [26–30], four in Australasia [21,22,30–32], seven in Europe [19,20,33–38] and five in North America [23–25,39–42]. Reported mean ages of participants ranged from 55–74 years. Nineteen trials included participants of both sexes; one did not report the gender of participants [40]. ‘Usual care’ was the comparator in 16 trials; however, in one six-month long intervention, control participants were provided with study information and educational materials at two-monthly intervals [39]. In a ‘telephone support’ trial, Damush et al., (2011) made placebo telephone calls that mimicked the intervention schedule [41]. Goldfinger et al., (2012) conducted risk factor assessments for control participants and made subsequent medical referrals, as appropriate [23]. Kono et al., (2013), in their exercise and education trial, provided control participants with usual care augmented by healthy lifestyle advice at baseline, 3 and 6 months [27].

Intervention duration ranged from ‘one off’ sessions to 6 weeks to 12 months. Five interventions comprised a discrete educational intervention with no follow-up (other than for data collection purpose) [22,23,29,31,36], one provided education and follow-up, in the form of counselling, at 3 and 6 months post-intervention [27]. Ten interventions comprised an initial session (e.g. assessment, education, information giving, counselling) followed by structured follow-up [31–35,38–42]. Two were monthly interventions with lasted for 3 months [19,30], and one computer-based intervention occurred on an individual basis [37]. Peng et al. [28] provided no details regarding intervention duration. Delivery methods included computer software, one-to-one sessions, and group work. Of the 19 interventions that specified delivery format, eight were delivered one-to-one [27,28,33–35,39,40,42]; four were delivered one-to-one and to a carer/family member [19,20,30–32], five used groups/workshops [21–24,26, 29,36]—one of these [23,24] used a peer-led model, the only example of peer-led delivery, and two used computer software to deliver intervention content [37,38].

Five studies reported overt family involvement/participation in the intervention [19,20, 26,31,32,38]; three studies reported involving family members, but only if this was required and/or desired by the stroke survivor [30,34,40].

Six of the twenty behavioral interventions reported that intervention design and/or delivery was informed by a psychological theory of behavior change i.e. Social Cognitive Theory [43] [26,33,41], the Health Belief Model [44] [21,22,30] and the Transtheoretical model [45] [34]. Other theories or approaches were also described including self-management [23,29,41], self-regulation [26], and self-education [27].

### Methodological Quality

Overall the risk of bias was high or unclear. Lack of detailed reporting resulted in many papers being appraised as ‘unclear’. Principal sources of bias were block randomisation, poor allocation concealment, lack of allocation blinding and selective outcome reporting particularly in relation to attrition. Sixteen papers reported data that could be used in meta-analysis. Of these, 10 performed ITT for all outcomes [20,22,25,27,30,33,34,36,38,39], 1 trial performed ITT for mortality and vascular outcomes but not for medical adherence [28], in 1 trial it was unclear what type of analysis was performed [26], and 4 trials did not use ITT analysis [32,35,37,42] (see Table 2).

**Table 2:** Risk of Bias.

	Random sequence generation (selection bias)	Allocation concealment (selection bias)	Blinding of assessors (performance bias)	Blinding of outcome assessment (detection bias) (patient reported outcomes)	Incomplete outcome data addressed (attrition bias)	Selective outcome reporting (reporting bias)	Other sources of bias i.e. baseline imbalance
Adie & James 2010	−	?	−	−	+	+	+
Allen et al., 2009	−	+	+	+	+	+	+
Banet & Felchlia 1997	?	?	?	?	?	?	?
Chanruengvanich et al., 2006	?	?	+	−	+	−	+
Damush et al., 2011	−	?	?	−	−	−	−
Eames et al., 2013	+	?	−	?	+	+	+
Ellis et al., 2005	+	+	+	−	+	+	+
Faulkner et al., 2013	+	+	?	+	+	+	+
Flemming et al., 2013	−	?	?	−	−	−	−
Gilham & Endacott 2010	+	+	?	−			−
Goldfinger et al., 2012	−	?	?	?	?	?	?
Hornnes et al., 2011	+	?	+	−	−	+	+
Joubert et al., 2006	−	−	−	−	−	−	+
Joubert et al., 2009	?	−	−	−	−	+	−
Kirk et al., 2013	?	?	−	+	+	+	+
Kono et al., 2013	+	+	?	+	+	+	+
Maasland et al., 2007	+	+	+	−	−	−	−
Peng et al., 2014	−	−	?	?	?	?	?
Sit et al., 2007	+	−	+	−	+	−	+
Wolfe et al., 2010	+	+	+	−	−	+	+

Key: + Low risk of bias;? Unclear risk of bias; − High risk of bias

### Meta-analyses: Physiological outcomes

Sufficient data were extracted to enable meta-analysis of a range of physiological outcomes of interest including blood pressure, blood lipids, and anthropomorphic measurements.

#### Blood pressure

**Fig 2:**
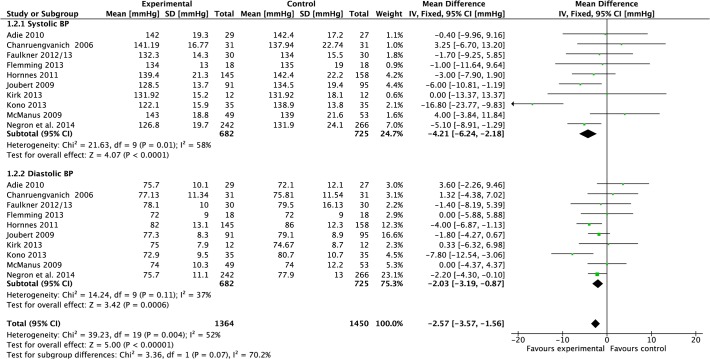
Forest Plot: Blood Pressure.

Data were pooled from 10 studies reporting blood pressure [20,22,25–27,32,33,35,36,42] (Fig. 2). Compared to control, meta-analysis demonstrated that the mean effect of intervention on systolic blood pressure post-treatment was a significant reduction of 4.21 mmHg (−6.24 to −2.18, P<0.0001, I^2^ = 58%, 1,407 participants), however, there was moderate heterogeneity. The mean effect on diastolic blood pressure was a significant reduction of 2.03 mmHg (−3.19 to −0.87, P = 0.006, I^2^ = 37%, 1,407 participants); heterogeneity was moderate.

#### Blood lipids

**Fig 3:**
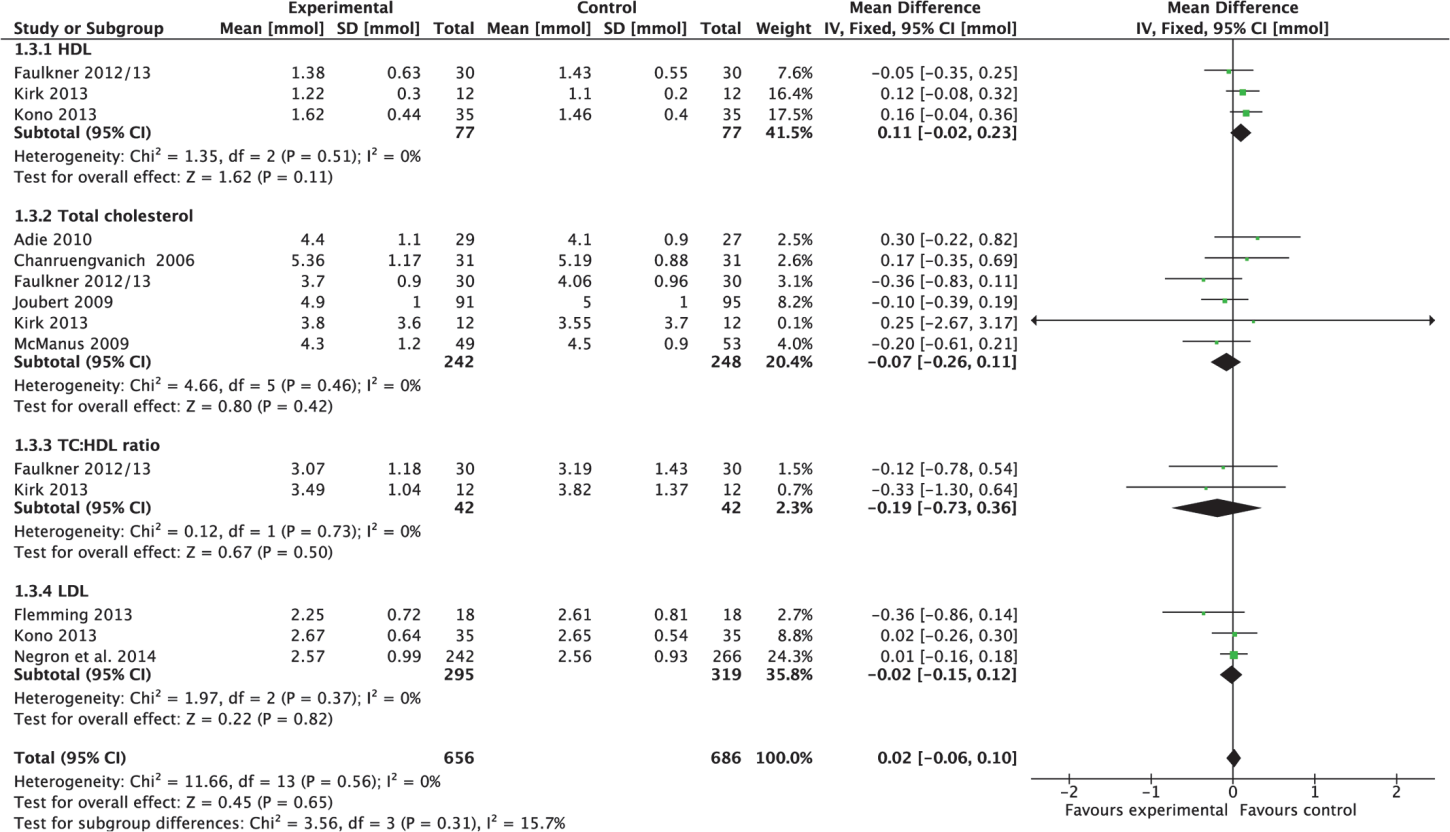
Forest Plot: Blood Lipids.

Data were pooled from three studies reporting HDL results [22,27,36]. Meta-analysis demonstrated that the mean effect of intervention was a non-significant increase of 0.11 mmol (−0.02 to 0.23, P = 0.11, I^2^ = 0%, 154 participants; Fig. 3). Data were pooled from three studies reporting LDL results [25,27,42]. Meta-analysis demonstrated that the mean effect of intervention was a non-significant reduction of 0.02 mmol (−0.15 to 0.12, P = 0.37, I^2^ = 0%, 614 participants; Fig. 3). Data were pooled from six studies reporting total cholesterol [20,22,26,32,33,36]. Meta-analysis demonstrated that there was a non-significant effect of intervention compared to control on total cholesterol post-treatment (−0.07 mmol, −0.26 to 0.11, P = 0.42, I^2^ = 0%, 490 participants; Fig. 3). Data were pooled from two studies reporting total cholesterol:HDL ratio [22,36]. Meta-analysis demonstrated that there was a non-significant effect of intervention compared to control post-treatment (−0.19 mmol, −0.73 to 0.36, P = 0.50, I^2^ = 0%, 84 participants; Fig. 3).

#### Other blood tests

**Fig 4:**
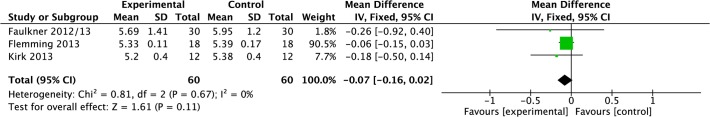
Forest Plot: Fasting Blood Glucose.

**Fig 5:**
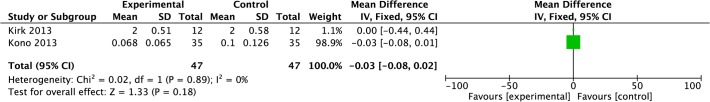
Forest Plot: High Sensitivity-CRP.

**Fig 6:**
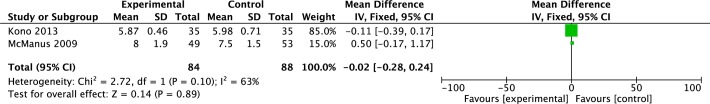
Forest Plot: HbA1c.

**Fig 7:**
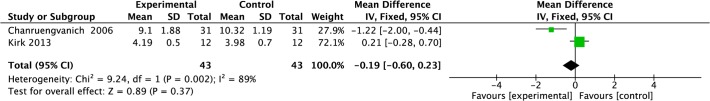
Forest Plot: Fibrinogen.

Data were pooled from three studies reporting fasting blood glucose [22,36,42]. Meta-analysis demonstrated that there was no significant effect of intervention compared to control post-treatment (−0.07 mmol/l, −0.16 to 0.02, P = 0.11, I^2^ = 0%, 120 participants; Fig. 4). Data were pooled from two studies reporting high sensitivity-CRP (a cardiac risk marker) [27,36]. Meta-analysis demonstrated that there was no significant effect of intervention compared to control post-treatment (−0.03 mg/l, −0.08 to 0.02, P = 0.18, I^2^ = 0%, 94 participants; Fig. 5). Data were pooled from two studies reporting HbA1c [20,27]. Meta-analysis demonstrated that there was no significant effect of intervention compared to control post-treatment (−0.02, −0.28 to 0.24, P = 0.89, I^2^ = 63%, 172 participants; Fig. 6), however, heterogeneity was high. Data were pooled from two studies reporting fibrinogen results [26,36]. Meta-analysis demonstrated that there was no significant effect of intervention compared to control post-treatment (−0.19 g/l, −0.60 to 0.23, P = 0.37, I^2^ = 89%, 86 participants; Fig. 7), however, heterogeneity was high.

#### Anthropomorphic measurements

**Fig 8:**
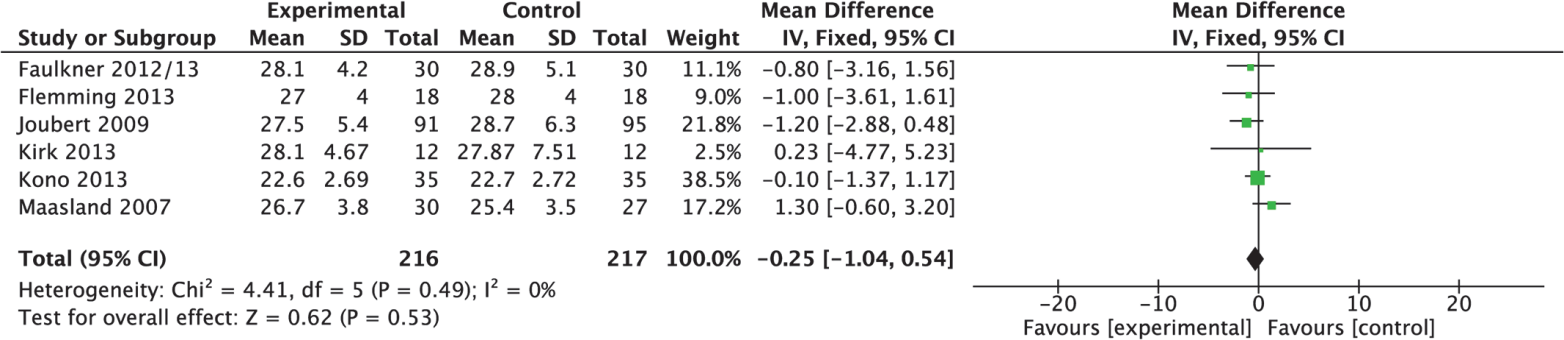
Forest Plot: Body Mass Index.

**Fig 9:**
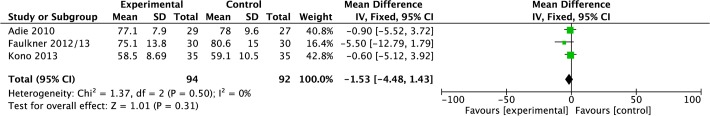
Forest Plot: Weight.

**Fig 10:**
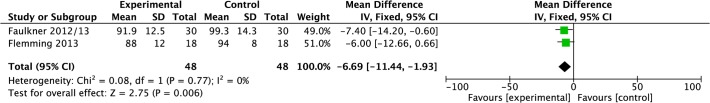
Forest Plot: Waist Circumference.

**Fig 11:**
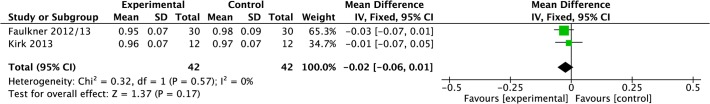
Forest Plot: Waist:Hip Ratio.

Data were pooled from six studies reporting total BMI results [22,27,32,36,37,42]. Meta-analysis demonstrated that post-treatment there was a non-significant reduction 0.25 kg/m^2^ (−1.04 to 0.54, P = 0.53, I^2^ = 0%, 433 participants; Fig. 8). Data were pooled from three studies reporting weight [22,27,33]. Meta-analysis demonstrated that post-treatment there was a non-significant reduction of −1.53 kg (−4.48 to 1.43, P = 0.31, I^2^ = 0%, 186 participants; Fig. 9). Data were pooled from two studies reporting waist circumference [22,36]. Meta-analysis demonstrated that post-treatment there was a significant reduction in waist circumference in intervention compared to control post-treatment (−6.69 cm, −11.44 to −1.93, P = 0.006, I^2^ = 0%, 96 participants; Fig. 10). Data were pooled from two studies reporting waist:hip ratio [22,36]. Meta-analysis demonstrated that post-treatment there was no significant effect of intervention compared to control post-treatment (−0.02 cm, −0.06 to 0.01, P = 0.17, I^2^ = 0%, 84 participants; Fig. 11).

### Meta-analyses: Lifestyle behavior outcomes

No meta-analyses were possible for physical activity or stress management due to the heterogeneity of the outcome measures used and the outcomes reported. However, meta-analyses were conducted for smoking status, diet, and medication compliance.

#### Smoking status

**Fig 12:**
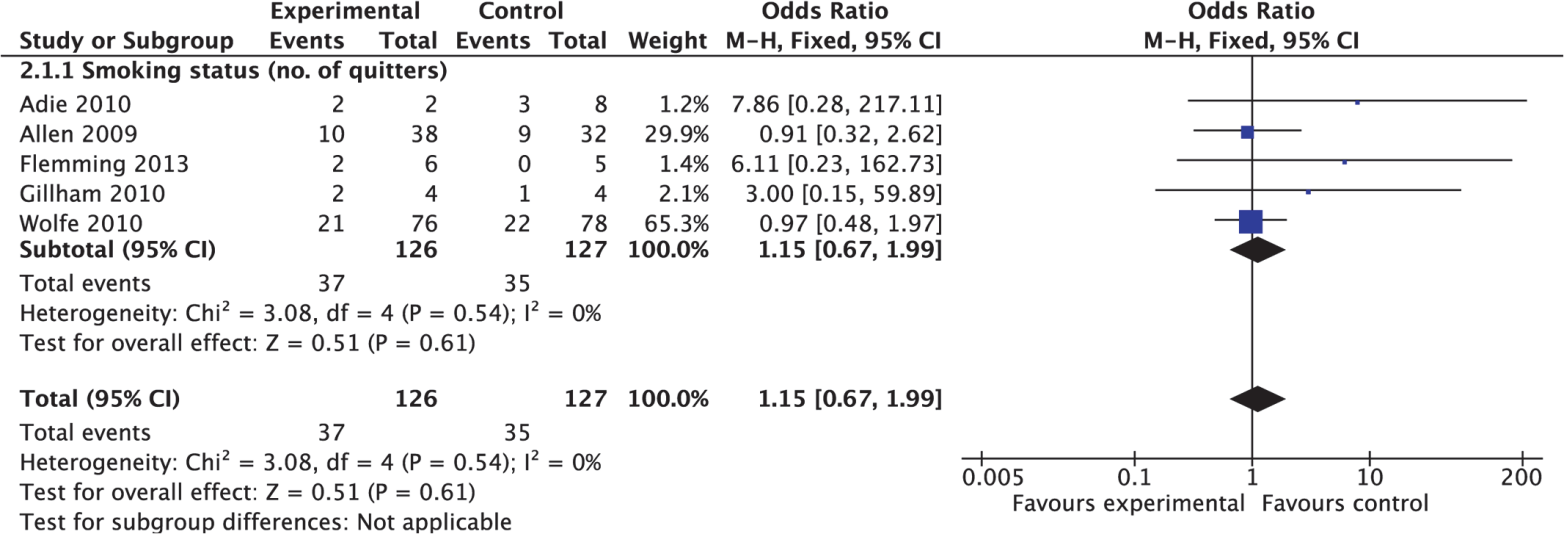
Forest Plot: Smoking.

Data were pooled from five studies reporting current smoking status post treatment [33,34,38,39,42]. Meta-analysis suggested there was no significant difference in odds of an individual being a current smoker post-treatment in intervention group compared to control (OR 1.15, 0.67 to 1.99, P = 0.61, I^2^ = 0%, 253 participants; Fig. 12). It should be noted that four studies reported similar smoking levels at baseline in both groups. However, in the study by Adie et al., (2010) over recruitment of smokers to the control group was acknowledged [33].

#### Diet

**Fig 13:**
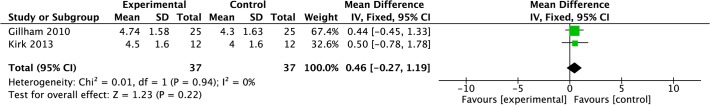
Forest Plot: Fruit and Vegetable Consumption.

Data were pooled from two studies reporting daily fruit and vegetable consumption [34,36]. Meta-analysis suggested there was no significant difference in fruit and vegetable consumption in intervention group compared to control (0.46, −0.27 to 1.19, P = 0.22, I^2^ = 0%, 74 participants; Fig. 13).

#### Medication compliance

**Fig 14:**
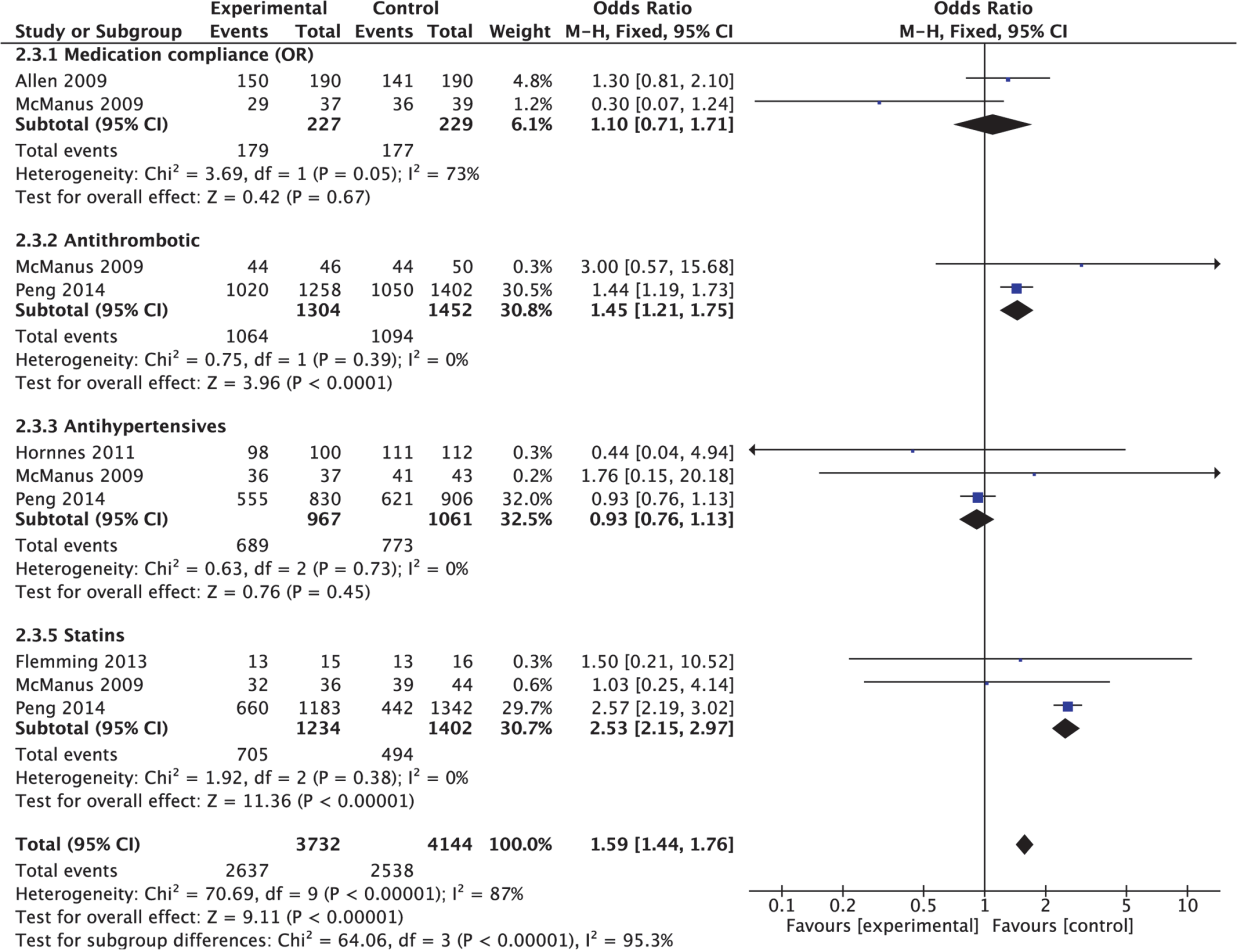
Forest Plot: Medication Compliance.

Data were pooled from two studies that had measured compliance with all relevant, prescribed medication [20,39]. Meta-analysis suggested there was no significant difference in odds of an individual complying with medication post-treatment in intervention group compared to control (OR 1.10, 0.71 to 1.71, P = 0.67, I^2^ = 73%, 456 participants; Fig. 14), with high heterogeneity. However, some studies reported compliance with specific medications and the findings varied. Data were pooled from two studies reporting compliance with antithrombotic medication [20,28]. Meta-analysis suggested there was a significant increase in odds of an individual complying with antithrombotic medication post-treatment in intervention group compared to control (OR 1.45, 1.21 to 1.75, P<0.0001, I^2^ = 0%, 2,792 participants; Fig. 14). Data were pooled from three studies reporting compliance with antihypertensive medication [20,28,35]. Meta-analysis suggested there was no significant difference in odds of an individual complying with antihypertensive medication post-treatment in intervention group compared to control (OR 0.93, 0.76 to 1.13, P = 0.45, I^2^ = 0%, 2,028 participants; Fig. 14). Data were pooled from three studies reporting compliance with statins [20,28,42]. Meta-analysis suggested there was a significant difference in odds of an individual complying with statin medication post-treatment in intervention group compared to control (OR 2.53, 2.15 to 2.97, P< 0.00001, I^2^ = 0%, 2636 participants; Fig. 14).

### Meta-analyses: Stroke knowledge

Due to the heterogeneity of the outcomes reported across the 20 studies and the heterogeneity of the outcome measures used, there were insufficient data relating to changes in stroke knowledge to allow meta-analysis.

### Meta-analyses: Psychosocial outcomes

**Fig 15:**
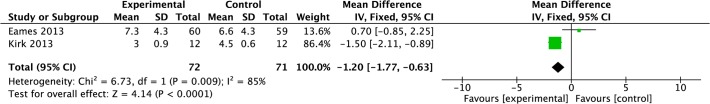
Forest Plot: Anxiety.

Sufficient data were extracted from two studies [30,36] to enable pooling of anxiety data, as measured using the Hospital Anxiety and Depression Scale [46]. Meta-analysis demonstrated that post-treatment there was a significant reduction in anxiety in the intervention group compared to control post-treatment (−1.20, −1.77 to −0.63, P<0.0001, I^2^ = 85%, 143 participants; Fig. 15), however, heterogeneity was high.

### Meta-analyses: Recurrent Events and Mortality

#### Recurrent TIA/Stroke

**Fig 16:**
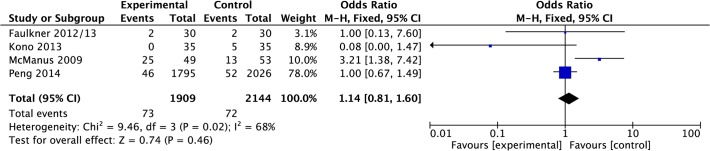
Forest Plot: TIA/Stroke Recurrence.

Data were pooled from four papers reporting recurrence of TIA/stroke events [20,22,27,28]. Meta-analysis suggested that there was no significant difference in odds of recurrence of TIA/stroke in the intervention group compared to control post-treatment (OR 1.14, 0.81 to 1.60, P = 0.46, I^2^ = 68%, 4,053 participants; Fig. 16), however, heterogeneity was high.

#### Recurrent cardiac events

**Fig 17:**
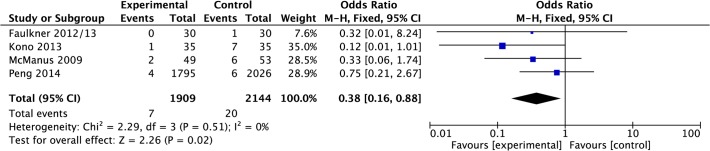
Forest Plot: Cardiac Events.

Data were pooled from four papers reporting recurrence of cardiac events [20,22,27,28]. Meta-analysis suggested that there was a significant reduction in odds of cardiac events in the intervention group compared to control post-treatment (OR 0.38, 0.16 to 0.88, P = 0.02, I^2^ = 0%, 4,053 participants; Fig. 17).

#### Mortality

**Fig 18:**
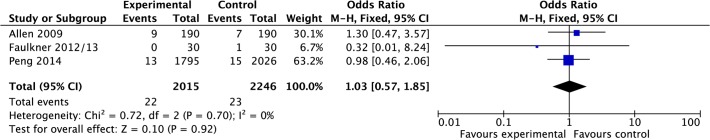
Forest Plot: Mortality.

Data were pooled from four papers reporting mortality [20,22,28,39]. Meta-analysis suggested that there was no significant difference in odds of death in the intervention group compared to control post-treatment (OR 0.91, 0.52 to 1.59, P = 0.74, I^2^ = 0%, 4,354 participants; Fig. 18).

### Meta-analyses: Long-term outcomes (sub-group analysis) 12-month data

In terms of long-term effects of intervention, the longest follow-up time for which there were sufficient data to enable meta-analyses was 12 months. Data from this time point were available for only two outcomes i.e. BP and BMI.

**Fig 19:**
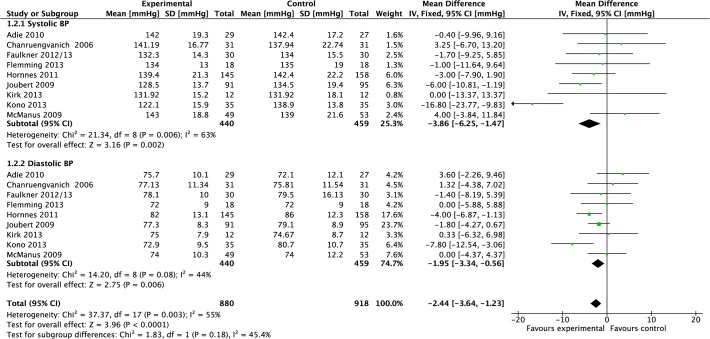
Forest Plot: 12-month Blood Pressure.

Data were pooled from three studies reporting blood pressure (systolic and diastolic) results at 12-months [32,35,42]. Compared to control, meta-analysis demonstrated that the mean effect of intervention on systolic blood pressure post-treatment was a significant reduction of 4.19 mmHg (−7.46 to −0.93, P = 0.01, I^2^ = 0%, 525 participants; Fig. 19). Compared to control, meta-analysis demonstrated that the mean effect of intervention on diastolic blood pressure post-treatment was a significant reduction of 2.49 mmHg (−4.27 to −0.70, P = 0.01, I^2^ = 0%, 525 participants; Fig. 19).

**Fig 20:**
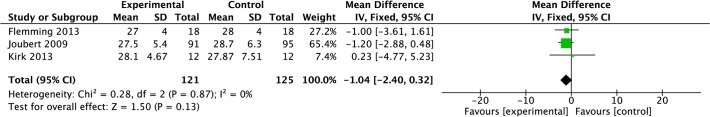
Forest Plot: 12-month Body Mass Index.

Data were pooled from three studies reporting BMI results at 12-months [32,36,42]. Compared to control, meta-analysis demonstrated that the mean effect of intervention on BMI post-treatment was a non-significant reduction of 1.04 kg (−2.40 to 0.32, P = 0.13, I^2^ = 0%, 246 participants; Fig. 20).

## Discussion

This systematic review and meta-analysis of multimodal interventions for the prevention of secondary stroke included 20 RCTs (6,373 participants). Where data were available, meta-analyses were conducted for physiological, lifestyle and psychosocial outcomes, and for recurrence and mortality. Meta-analysis showed a significant effect of intervention on the reduction of systolic and diastolic blood pressure; these results had large numbers of participants. Positive trends were noted in relation to blood lipids and anthropomorphic measures. In terms of lifestyle factors, there was significant positive effect of intervention on medication compliance, specifically for antithrombotic and statins, and both with large numbers of participants. A significant positive effect was demonstrated in relation to anxiety, as measured by HADS; however participant numbers were small, and this result should be treated with caution. And, although meta-analysis demonstrated no significant effect of intervention on mortality or recurrence of stroke/TIA, a significant reduction in recurrence of cardiac events was noted.

The review included only studies published in the English language and therefore may have excluded other potentially relevant studies. And, as with any systematic literature review, it is possible that the searches did not identify all relevant English language studies, however the searches were extensive and included a comprehensive grey literature search strategy. A detailed definition of ‘multimodal intervention’ was applied, however the studies included in the review varied considerably in many respects, e.g. terms of length of intervention and follow up, format, time post-stroke. This heterogeneity was not accounted for in the analyses, although the 12-month follow-up data indicate that the effect of intervention persists beyond the short-term. Meta-analysis was possible for only a limited range of outcomes of interest. There were several reasons for this: lack of consistency in measures used across the studies; lack of use of/availability of standardised outcomes measures, particularly in relation to behavioral outcomes; lack of consistency of reporting across the studies e.g. mean and deviation or change; and selective reporting i.e. not reporting or making available the results of outcomes for which data were collected. As described in the results section, data from only 16 of papers were included in the various meta-analyses. Of these 1 trial performed ITT for mortality and vascular outcomes but not for medical adherence, in 1 trial it was unclear what type of analysis was performed, and 4 trials did not use ITT analysis. Although we acknowledge that poorer quality studies i.e. studies that do not report ITT data, may introduce bias to the results, sensitivity analysis could not be performed due to the small number of trials in some analysis. Therefore, if had we excluded trials based on quality, some analysis would not have been possible due to lack of data. A further limitation of the studies included in the review was the lack of completeness of intervention description. Such lack of detail prohibits replication or development of the intervention by subsequent researchers and/or clinicians thus wasting already limited research resources [47].

Due to the paucity of available data, all relevant studies were included, irrespective of methodological quality. Most of the studies in this review rated poorly, however this, in part, reflects some Risk of Bias criteria that, with hindsight, are not best suited to the appraisal of RCTs reporting behavioral interventions. For example, behavioral studies often rely on self-report to measure outcomes, and participants and interventionists cannot be blinded to the intervention.

Meta-analysis showed a significant effect of intervention on reduction of blood pressure. As indicated above, these analyses had large participant numbers and therefore are of worthy of note. Controlling blood pressure to within recommended parameters (<140/85mmHg; 130/80 mmHg for people with diabetes) may reduce the risk of stroke by approximately 40% [48,49]; therefore controlling blood pressure represents an important secondary prevention outcome target.

In terms of blood lipids, meta-analysis demonstrated a non-significant reduction in total cholesterol as a result of intervention; a finding echoed by Lennon et al., (2013) in their review of lifestyle interventions, who noted that a lack of detailed reporting of data regarding full lipid profiles excludes the possibility of looking at the effect of intervention on HDL ratio, which is a more sensitive indicator of risk and risk reduction [13]. Contemporary guidelines suggest that reduction of total cholesterol with a statin, reduces the relative risk of ischaemic stroke to 0.8 (0.70 to 0.92), indicating the importance of cholesterol as a secondary prevention outcome target, following TIA and ischaemic stroke [49].

In terms of lifestyle behavior, all included studies addressed smoking cessation, as might be expected given the long-established importance of tobacco use as an independent risk factor for stroke [6,50,51]. However, data from only five studies [33,34,38,39,42] were included in the meta-analysis, which demonstrated no significant positive effect of intervention on smoking status. All five interventions provided education and/or advice about smoking as a lifestyle risk factor for stroke, and some made use of motivational interviewing or counselling approaches; however, it was not clear, from what was reported, whether patients had been advised to use pharmacotherapy (e.g. nicotine replacement therapy) to support a cessation attempt. This is important as clinical guidelines suggest that smoking cessation interventions should include both behavioral support and pharmacotherapy [52]. Again, while advice and “support” were mentioned, there was little detail of what this actually meant. Brief smoking cessation interventions are an important first step in the chain of support known to be effective in assisting cessation; however, more intensive support provided by cessation specialists is known to be the most effective strategy in supporting an actual quit attempt [52]. In the UK it is recommended that patients wishing to stop smoking be referred to a network of specially trained advisors [52]. As discussed, little was written about the smoking cessation element of the various interventions and, similar to Lennon et al., (2013), we are concerned that the importance of the smoking cessation message, and the need for pharmacotherapy and more intensive support to assist a cessation attempt, may become lost in a multi-modal secondary prevention intervention [13]. Finally, all but one of the studies relied on self-report of smoking status. The Russell Standards recommend that all trials reporting on the effectiveness of smoking cessation interventions use objective measures of smoking status such as serum or salivary cotinine and/or expired carbon monoxide levels.

In terms of outcomes associated with lifestyle, meta-analysis for medication compliance demonstrated significant positive effect of intervention. As indicated above, these meta-analyses included large participant numbers, rendering these findings of greater interest to clinicians and researchers. Optimal medication compliance is vital in the prevention of recurrent strokes and other cardiovascular events [49]. However, the review found evidence of selective medication compliance i.e. participants were compliant with antithrombotics and statins but not with antihypertensives. Recent work by O’Carroll et al., (2013) found that predictors of poor adherence (unintentional and intentional) to medication regimes included reduced cognitive function [53], a common consequence of stroke known to be associated with poor medication adherence, and treatment beliefs i.e. perceived benefit versus perceived risk (side effects). Allen et al., (2009) attempted to address unintentional non-compliance by providing pill organisers and pre-packaged medication systems, as appropriate, and both Allen et al., (2009) and Hornnes et al., (2011) in intensive, nurse-led interventions provided education regarding medication and the importance of compliance, with mixed effect [39,35]. However, O’Carroll et al., (2013) found that a brief intervention addressing erroneous beliefs about medication and stroke improved medication adherence by 10% in a population of older adults following stroke [53].

Data pooled from two small (n = 143 participants) studies [30,36] indicated that intervention helped reduce anxiety, as measured by HADS. Although this is a positive outcome, the small number of participants means it should be treated with caution. The lack of reporting of psychosocial outcomes highlights need for further work in this area. In spite of the prevalence of psychological consequences of stroke, there are few trials of effectiveness of behavioral interventions designed to address psychosocial issues after stroke. For example, despite widespread recognition of mindfulness as a therapeutic intervention, a recent review of mindfulness-based interventions following TIA/stroke identified only four studies, and these were of poor methodological quality [54].

No meta-analyses were possible for any learning outcomes. Although, interventionists acknowledged the important role of education and knowledge acquisition in relation to behavior change, only four reported knowledge outcomes, and these used heterogeneous measures of acquired knowledge. Knowledge, together with skills acquisition, is understood to have a role as a precursor to behavior change, and education has been identified as a ‘source of behaviour’ [55]. Our qualitative review of participants’ perceptions of secondary prevention interventions described acquisition of stroke-specific knowledge as a factor contributing to the development of confidence [56], which is in turn a necessary precursor to engaging in and sustaining positive lifestyle behavior change [57]. Joubert et al., (2009) noted that cholesterol levels were reduced in participants who recalled receiving advice, and that this was statistically significant (P = 0.005) [32]. Based on this evidence, they suggest that ‘*advice translated into risk-factor modification’* (p.282), although why this effect was demonstrated for cholesterol only, is unclear [32].

In terms of recurrence, there was a significant reduction in the recurrence cardiac events, but not in reduction of the odds of TIA/stroke recurrence. The large numbers include in the meta-analysis give weight to this significant finding. Given the commonalities in underlying risk, this is perhaps an unexpected finding; however, it may reflect higher precision in measuring and reporting cardiac events. In two studies the process for recording recurrent events is unclear [22,28] and one study relied on self-report, acknowledging this as a limitation [20]. Only Kono et al., (2013) made efforts to verify recurrent events clinically [27]. Alternatively, it might be argued that reduction in cardiac outcomes, rather than TIA/stroke outcomes is not too surprising, as cardiac outcomes may be physiologically more amenable to secondary prevention than stroke. Also, improved compliance with statins and antithrombotic medication (rather than antihypertensives) is likely to favour cardiac prevention. However, this hypothesis is tentative and should be regarded with caution.

Meta-analysis demonstrated no significant effect of intervention on mortality, perhaps an unsurprising finding given the short follow up period reported in most papers. Evidence regarding effectiveness in terms of recurrence of stroke and other vascular events, and stroke mortality, will only be generated by long-term follow-up of large cohorts; this review highlights the need for such studies.

The limited effectiveness of the few studies that have been conducted may reflect essential flaws in intervention design. In particular, a lack of theoretical underpinning and the failure to draw on powerful family dynamics [58,59]. The lack of appropriate theory underpinning behavior change is surprising. Intervening to effect behavior change in the general population is complex and challenging. Following stroke, residual impairments may vary in intensity and complexity, and may present considerable barriers to engaging with lifestyle behavior change, including formal, multimodal interventions. Evidence suggests that behavioral interventions are more likely to be effective, across a broader range of outcomes, if they are grounded in behavior change theory and are delivered by healthcare professionals with appropriate training [60]. In terms of family theory, a growing body of literature supports the active involvement of family members in nursing interventions [61] and in post-stroke rehabilitation in particular [62,63]. However, none of the studies in this review were overtly family-centred, only involving a family member if required to support the participant in some aspect of the intervention. In the cardiac field, where multimodal secondary prevention programmes are well established, Euroaction, is an example of an overtly family-centred intervention that reduced the risk of cardiovascular disease in families, who together made healthier food choices and became more physically active [64].

## Conclusions

In summary, there is growing evidence of the effectiveness of multimodal interventions following TIA and stroke particularly in relation to achieving blood pressure reduction, medication compliance and anxiety reduction. The findings from this review are complex and should be interpreted with caution. They relate only to intervention outcomes and not to processes and mechanisms of action. Future, large-scale trials RCTs should measure and report a wider range of relevant outcomes, across the domains described here i.e. physiological, behavioral, psychosocial, learning, and recurrence/mortality, using standardised measures validated with stroke populations. Interventionists should provide greater detail regarding intervention design, delivery and fidelity, so that the essential work to understand the processes and mechanisms of action can be undertaken.

## Supporting Information

S1 PRISMA Checklist (DOCX)

S1 Box Participants, Interventions, Comparator and Outcomes (PICO). (DOCX)

S1 Text PROSPERO-registered review protocol. (PDF)

S2 Text Search strategy finalised for use in MEDLINE. (DOCX)

S3 Text Data extraction tool. (DOCX)
