# Supplementary material for: Multimodal Secondary Prevention Behavioral Interventions for TIA and Stroke: A Systematic Review and Meta-Analysis
Source: PLoS One. 2015 Mar 20;10(3):e0120902. doi: 10.1371/journal.pone.0120902 (PMC4368743; doi:10.1371/journal.pone.0120902)
Supplement: S1 Box — (DOCX) [file pone.0120902.s002.docx]

**Box S1 Participants, Interventions, Comparator and Outcomes (PICO)**

|  | **Inclusion criteria** | **Exclusion criteria** |
| --- | --- | --- |
| **Population** | Adults (aged ≥18 years)  Stroke i.e. ischaemic stroke, haemorrhagic stroke, subarachnoid haemorrhage, minor stroke, transient ischaemic attack  Mixed populations where stroke data can be extracted | Other diseases/conditions |
| **Intervention** | Multimodal lifestyle behaviour change interventions designed to address the prevention of recurrent stroke  Note: Multimodal is defined as an intervention which comprises 3 components: 1) medication education and/or medication compliance education; 2) education or active* information provision; and 3) one or more ‘behaviours’ i.e. tobacco use, alcohol consumption, physical activity, diet and compliance with stress management strategies and medication compliance  * An intervention is classified as active if, *following the provision of the information, there was a purposeful attempt to allow the participant to assimilate the information and a subsequent agreed plan for clarification and consolidation or reinforcement. e.g. education sessions followed by question and answer sessions and follow-up to check understanding* (Forster et al., 2012) | Passive information giving/advice  Public health/primary prevention initiatives and interventions  Pharmacotherapy (as sole focus) |
| **Comparator** | Usual care, or modified usual care e.g. a schedule of phone calls which mimics the schedule of ‘intervention’ calls made to the intervention group [41] | Other ‘active’ interventions |
| **Outcomes** | *Primary outcomes*  Physiological outcomes e.g. blood pressure, blood lipids  Behaviour change i.e. change *re* tobacco use, alcohol consumption, diet, physical activity, medication compliance  *Secondary outcomes*  Psychosocial outcomes: e.g. anxiety  Learning outcomes e.g. stroke knowledge  Recurrence: incidence of recurrent stroke and other vascular events amongst the study population | Public health outcomes  Primary prevention  Functional/motor rehabilitation outcomes  QoL (as sole focus)  Functional/motor rehabilitation outcomes plus QoL  Return to employment  Social functioning/participation (as sole focus)  Exercise (tolerance) testing  Aerobic capacity/lung function |

Forster et al., Information provision for stroke patients and their caregivers. Cochrane Database of Systematic Reviews 2012, Issue 11. Art. No.: CD001919
