# Supplementary material for: Multimodal Secondary Prevention Behavioral Interventions for TIA and Stroke: A Systematic Review and Meta-Analysis
Source: PLoS One. 2015 Mar 20;10(3):e0120902. doi: 10.1371/journal.pone.0120902 (PMC4368743; doi:10.1371/journal.pone.0120902)
Supplement: S1 Text — (PDF) [file pone.0120902.s003.pdf]

Secondary prevention lifestyle interventions for transient ischaemic attack, stroke: a systematic review

Maggie Lawrence, Jan Pringle, Jo Booth, Susan Kerr

Citation

Maggie Lawrence, Jan Pringle, Jo Booth, Susan Kerr. Secondary prevention lifestyle interventions for transient ischaemic attack, stroke: a systematic review. PROSPERO 2012:CRD42012002538 Available from http://www.crd.york.ac.uk/PROSPERO_REBRANDING/display_record.asp?ID=CRD42012002538

Review question(s)

Review objectives:

1) To identify and critically appraise RCTs reporting secondary prevention lifestyle interventions for TIA/stroke.

2) To extract data from quantitative studies and meta-analyse, where possible.

3) To extract and collate data regarding educational content and behavioural change techniques and strategies.

Searches

Databases: AMED, ASSIA, Cochrane Systematic Reviews, CINAHL, DARE, DORIS, EMBASE, ERIC, HMIC, MEDLINE, PsycINFO, Social Services Abstracts

Grey literature sources: EthOS, Zetoc Conference Proceedings, pharmaceutical companies, key professionals, voluntary organisations

Restricted to: English language; years 1980-2014 (January)

Types of study to be included

Inclusion criteria:

randomised controlled trials

Exclusion criteria:

Epidemiological studies

Audit

Literature reviews

Clinical guidelines

Discussion/opinion papers

Condition or domain being studied

TIA/stroke secondary prevention, with particular reference to modifiable lifestyle factors.

Participants/ population

Inclusion population:

Adults (>/= 18 years);

stroke (i.e. ischaemic stroke, haemorrhagic stroke, subarachnoid haemorrhage, minor stroke, transient ischaemic attack).

Exclusions:

other diseases/conditions;

carers/family members/family caregivers (as sole focus);

health professionals (as sole focus).

Intervention(s), exposure(s)
Inclusion criteria:

Lifestyle interventions designed to address the prevention of TIA/Stroke recurrence

Multimodal (i.e. pharmacotherapy, education, and at least one lifestyle risk factor).

Exclusion criteria:

Public health/primary prevention initiatives and interventions;

Pharmacotherapy (as sole focus).

Comparator(s)/ control
All comparators/controls will be considered as part of the review.

Context
Studies will be sought and included from any relevant setting.

Outcome(s)
Primary outcomes
Primary outcome measures:

Lifestyle: behaviour change re tobacco use, alcohol consumption, diet, physical activity; psychosocial stress, medication compliance.

Physiological outcomes: e.g. blood pressure, blood sugar, blood lipids, weight, Body Mass Index, waist-hip ratio, waist circumference.

Secondary outcomes
Secondary outcome measures:

Psychological outcomes: wellbeing; life satisfaction; Quality of Life (QoL); self-rated health.

Learning outcomes: knowledge of: signs and symptoms of stroke, appropriate response, risk factors; health-related attitudes &/or beliefs.

Cardiovascular recurrence: incidence of recurrent cardiovascular events.

Data extraction, (selection and coding)
Quantitative data will be extracted from papers included in the review using a review-specific data extraction tool. Data extracted will include details regarding study methods, participant characteristics, intervention content, and outcomes.

Four researchers will be involved in data extraction, with discrepancies being resolved through team discussion and consensus.

Risk of bias (quality) assessment

Risk of Bias

Cochrane Collaboration Risk of Bias tool will be used.

Strategy for data synthesis

Data analysis

Data synthesis

Quantitative data will be pooled, where possible, in statistical meta-analysis. All results will be subject to double data entry. Odds ratio (for categorical data) and weighted mean differences (for continuous data) and their 95% confidence intervals will be calculated for analysis. Heterogeneity will be assessed using standard Chi-squared analyses. Where statistical pooling is not possible, findings will be presented in narrative form.

Analysis of subgroups or subsets

12-month data

Dissemination plans

Dissemination: results will be disseminated through local, national and international publications and conferences, and through the (UK) Stroke Association.

Contact details for further information

maggie lawrence

A256 Govan Mbeki Building

Glasgow Caledonian University

G4 0BA

margaret.lawrence@gcu.ac.uk

Organisational affiliation of the review

Glasgow Caledonian University

www.gcu.ac.uk

Review team

Dr Maggie Lawrence, Glasgow Caledonian University
Dr Jan Pringle, University of Dundee
Dr Jo Booth, Glasgow Caledonian University
Dr Susan Kerr, Glasgow Caledonian University

Collaborators

Professor Martin Dennis, University of Edinburgh

Anticipated or actual start date

01 May 2012

Anticipated completion date

30 June 2014

Funding sources/sponsors

The Stroke Association

Conflicts of interest
None known

Language
English

Country
Scotland

Subject index terms status
Subject indexing assigned by CRD

Subject index terms
Acute Coronary Syndrome; Health Behavior; Humans; Ischemic Attack, Transient; Life Style; Secondary Prevention; Stroke

Stage of review
Completed but not published

Date of registration in PROSPERO
10 August 2012

Date of publication of this revision
17 June 2014

Stage of review at time of this submission	Started	Completed
Preliminary searches	Yes	Yes
Piloting of the study selection process	Yes	Yes
Formal screening of search results against eligibility criteria	Yes	Yes
Data extraction	Yes	Yes
Risk of bias (quality) assessment	Yes	Yes
Data analysis	Yes	Yes

PROSPERO
International prospective register of systematic reviews
The information in this record has been provided by the named contact for this review. CRD has accepted this information in good faith and registered the review in PROSPERO. CRD bears no responsibility or liability for the content of this registration record, any associated files or external websites.
