# Supplementary material for: Multimodal Secondary Prevention Behavioral Interventions for TIA and Stroke: A Systematic Review and Meta-Analysis
Source: PLoS One. 2015 Mar 20;10(3):e0120902. doi: 10.1371/journal.pone.0120902 (PMC4368743; doi:10.1371/journal.pone.0120902)
Supplement: S2 Text — (DOCX) [file pone.0120902.s004.docx]

**Text S2 Search finalized for use in MEDLINE.**

1. exp Arteriosclerosis/

2. exp Basal Ganglia Cerebrovascular Disease/

3. exp Brain Infarction/

4. exp Brain Ischemia/

5. exp Carotid Artery Diseases/

6. exp Cerebral Hemorrhage/

7. exp Cerebral Infarction/

8. exp Cerebrovascular Disorders/

9. exp Coronary Artery Disease/

10. exp Coronary Disease/

11. exp Coronary Thrombosis/

12. exp Intracranial Arterial Diseases/

13. exp Intracranial Arteriovenous Malformations/

14. exp "Intracranial Embolism and Thrombosis"/

15. exp Intracranial Hemorrhages/

16. exp Ischemic Attack, Transient/

17. exp Myocardial Infarction/

18. exp Myocardial Ischemia/

19. exp Stroke/

20. exp Vasospasm, Intracranial/

21. exp Vertebral Artery Dissection/

22. 1 or 2 or 3 or 4 or 5 or 6 or 7 or 8 or 9 or 10 or 11 or 12 or 13 or 14 or 15 or 16 or 17 or 18 or 19 or 20 or 21

23. apoplex$.mp.

24. arter?osclerosis.mp.

25. brain infarct$.mp.

26. brain isch?emi$.mp.

27. (cerebral adj (haemorrhage or hemorrhage)).mp.

28. cerebral infarction.mp.

29. cerebrovascular accident.mp.

30. cerebrovascular disorder$.mp.

31. coronary arter?osclerosis.mp.

32. coronary artery disease.mp.

33. coronary disease.mp.

34. coronary thromb$.mp.

35. CVA.mp.

36. ((hemorrhagic or haemorrhagic) adj stroke).mp.

37. heart infarc$.mp.

38. "intracranial embolism and thrombosis".mp.

39. (intracranial adj (hemorrhag$ or haemorrhag$)).mp.

40. (isch?emi$ adj (heart or stroke)).mp.

41. transient isch?emic attack.mp.

42. myocard$ infarct$.mp.

43. myocard$ isch?emia.mp.

44. (post stroke or post-stroke or poststroke).mp.

45. stroke.mp.

46. TIA.mp.

47. 23 or 24 or 25 or 26 or 27 or 28 or 29 or 30 or 31 or 32 or 33 or 34 or 35 or 36 or 37 or 38 or 39 or 40 or 41 or 42 or 43 or 44 or 45 or 46

48. 22 or 47

49. exp Counseling/

50. exp Diet Therapy/

51. exp Exercise Therapy/

52. exp Health Education/

53. exp Health Promotion/

54. exp Nutrition Therapy/

55. exp Secondary Prevention/

56. exp Weight Reduction Programs/

57. 49 or 50 or 51 or 52 or 53 or 54 or 55 or 56

58. cardiac rehab$.mp.

59. counsel$.mp.

60. (exercise adj3 (intervent$ or program$ or educat$ or behavio$ or promotion$)).mp.

61. (health adj3 (intervent$ or program$ or educat$ or behavio$ or promotion$)).mp.

62. ((lifestyle or life style) adj3 (intervent$ or program$ or educat$ or behavio$ or promotion$ or advice or alter$ or chang$)).mp.

63. (multifactor$ adj3 (intervent$ or program$)).mp.

64. (multimodal adj3 (intervent$ or program$)).mp.

65. (patient adj3 (intervent$ or program$ or educat$)).mp.

66. risk reduction.mp.

67. (secondary adj3 (prevent$ or intervent$)).mp.

68. 58 or 59 or 60 or 61 or 62 or 63 or 64 or 65 or 66 or 67

69. 57 or 68

70. exp Alcohol Drinking/

71. exp Diabetic Diet/

72. exp Diet, Fat-Restricted/

73. exp Diet, Mediterranean/

74. exp Diet, Reducing/

75. exp Exercise/

76. exp Smoking/

77. exp Smoking Cessation/

78. exp Tobacco/

79. exp Stress, Psychological/

80. exp "Tobacco Use Cessation"/

81. exp Tobacco, Smokeless/

82. 70 or 71 or 72 or 73 or 74 or 75 or 76 or 77 or 78 or 79 or 80 or 81

83. (alcohol adj3 drinking).mp.

84. (diet$ adj3 (fat-restricted or Mediterranean or reducing or diabetic)).mp.

85. exercise.mp.

86. physical exercise.mp.

87. smoking.mp.

88. (stress adj3 psychological).mp.

89. tobacco.mp.

90. ((tobacco or smoking) adj3 cessation).mp.

91. (tobacco adj3 chew$).mp.

92. 83 or 84 or 85 or 86 or 87 or 88 or 89 or 90 or 91

93. 82 or 92

94. 48 and 69 and 93

95. limit 94 to (humans and yr="1980 -Current" and "all adult (19 plus years)" and humans)
