# Supplementary material for: Multimodal Secondary Prevention Behavioral Interventions for TIA and Stroke: A Systematic Review and Meta-Analysis
Source: PLoS One. 2015 Mar 20;10(3):e0120902. doi: 10.1371/journal.pone.0120902 (PMC4368743; doi:10.1371/journal.pone.0120902)
Supplement: S3 Text — (DOCX) [file pone.0120902.s005.docx]

**Text S3 Data Extraction Tool**

| **Bibliographic details** | | | | | |
| --- | --- | --- | --- | --- | --- |
| Author |  | Quality appraisal score | |  | |
| Year: Publication |  | | | | |
| Year: Data Collection |  | | | | |
| Country |  | | | | |
| Bibliographic Ref |  | | | | |
| References identified from reference list | yes/no  If yes, please provide details: | | Reviewer (initials) | |  |

| Study design |  | | | |
| --- | --- | --- | --- | --- |
| Study Aim |  | | | |
| Inclusion criteria |  | | | |
| Exclusion criteria |  | | | |
| **Baseline characteristics of study sample** | | | | |
|  | | Intervention group | Control /comparison group | |
| Sample size | |  |  | |
| Recruited from where? | |  | | |
| Gender | |  |  | |
| Age | |  |  | |
| Ethnicity | |  |  | |
| Socioeconomic status | |  |  | |
| Employment status | |  |  | |
| Marital status | |  |  | |
| Educational status | |  |  | |
| Living Arrangements | |  |  | |
| Co-morbidities (specify) | |  |  | |
| Lifestyle risk factors | | tobacco:  alcohol:  diet:  physical activity:  stress: | | tobacco:  alcohol:  diet:  physical activity:  stress: |
| Time between diagnosis and participation in the intervention | |  | | |
| Other (specify) | |  | | |
| *Stroke* | | | | |
| Definition | |  | | |
| Type e.g. ischaemic | |  |  | |
| Lesion location | |  |  | |
| Severity e.g. NIHSS | |  | | |
| Aphasia (number of participants; severity) | |  | | |
| Cognitive impairment (number of participants; severity) | |  | | |
| Affective disorders (number of participants; severity) | |  | | |

| Secondary Prevention Intervention | |
| --- | --- |
| Underpinning theory |  |
| Behaviour change taxonomy (Record relevant checklist numbers) |  |
| Intervention |  |
| Course content |  |
| Duration & frequency |  |
| Format: e.g. group |  |
| Who delivered the intervention?  (Record, profession, qualification and/or expertise as appropriate) |  |
| Did they have specific training? |  |
| No. of participants per group |  |
| Intervention materials |  |
| Intervention location |  |
| Cost to participants |  |
| Cost effectiveness |  |
| Transport issues |  |
| Gifts or honoraria |  |
| Family involvement |  |
| Website/associated resource(s) |  |
| Delivery fidelity |  |
| Other (specify) |  |
|  | |
| Intervention for control group | |
|  | |
|  | |
|  | |
|  | |
|  | |
|  | |

| Outcomes measurement | | |
| --- | --- | --- |
|  | Intervention group | Control /comparison group |
| Standardised outcomes measures (specify) |  | |
| Data collected by |  | |
| Study-specific outcomes measures (provide details) |  | |
| Other outcomes measured (provide details) |  | |
| No. of data collection time points |  | |
| Other (specify) |  | |

| Results | |
| --- | --- |
|  | |
| Limitations as noted by the authors |  |
| Authors’ recommendations |  |
| Authors’ conclusions |  |
| Reviewer’s comments |  |
